# The dogma of cetuximab in advanced squamous cell carcinoma of the head and neck after failure of surgery and radiotherapy: is it true among patients in upper Egypt?

**DOI:** 10.3332/ecancer.2023.1611

**Published:** 2023-10-09

**Authors:** Amal Rayan, Mohammed S Shahine, Khalid Rezk, Asmaa M Zahran, Mohamed Modather Aboshanif, Doaa A Gamal

**Affiliations:** 1 Clinical Oncology Department, Faculty of Medicine, Assiut University, Assiut 71515, Egypt; 2 Maxillofacial Surgery, General Surgery Department, Faculty of Medicine, Assiut University, Assiut 71515, Egypt; 3 Surgical Oncology Department, South Egypt Cancer Institute, Assiut University, Assiut 71515, Egypt; 4 Clinical Pathology Department, South Egypt Cancer Institute, Assiut University, Assiut 71515, Egypt; 5 Otorhinolaryngology Department, Faculty of Medicine, Assiut University, Assiut 71515, Egypt; a https://orcid.org/0000-0001-5995-9177

**Keywords:** squamous cell carcinoma of head and neck, cetuximab, recurrence, progression-free survival

## Abstract

**Background and aim:**

We aimed from the current study to explore the treatment results of cetuximab in combination with a weekly carboplatin and paclitaxel regimen in advanced squamous cell carcinoma of head and neck (HNSCC) after failure of radiotherapy and chemotherapy.

**Methods:**

This study was a non-randomised, single arm, phase 2 efficacy study conducted in two oncology centres in upper Egypt, we recruited 31 patients with recurrent HNSCC previously treated with concurrent chemoradiation ± surgery to receive weekly cetuximab, carboplatin and paclitaxel for 18 weeks followed by maintenance cetuximab every 2 weeks for 12 months. All patients underwent intention to treat analysis.

**Results:**

The current study revealed a significant reduction of the size of recurrent primary lesion (*p* < 0.001), without comparable significant reduction of regional lymph nodes (LNs) (*p* = 0.06), the current overall response rate (ORR) was 83.9%, ≥1-year progression-free survival (PFS) was 58.1%, also surgical intervention was succeeded to salvage 32.3% who did not achieve complete response to the current protocol, the median PFS was 12 months which was significantly affected by tumour site (*p* = 0.012), programmed death ligand-1 (PDL-1) expression (*p* = 0.01) and overall response rate (ORR) (*p* < 0.001).

**Conclusion:**

Based on favourable treatment outcomes, including high ORR and disease control rate, improved median PFS and tolerable toxicity profile, the current weekly cetuximab, carboplatin and paclitaxel with 1 year maintenance cetuximab in responding patients is considered a feasible and effective regimen.

## Introduction

Squamous cell carcinoma (SCC) of head and neck (HNSCC) is a common neoplasm with a rising incidence worldwide [1], globally, about 8% of newly diagnosed cases of cancers were of head and neck SCC and responsible for 10.2% of cancer deaths. In Egypt, head and neck cancer, predominantly squamous cell cancer, represented about 2.68% of all cancer burden and was responsible for 2.22% of all cancer deaths [2]. This type of cancer is commonly diagnosed in locally advanced stage with subsequent limited surgical opportunity and high morbidities associated with extensive surgery, in addition to poor general condition of the diseased patients, all these challenges necessitate new treatment approaches away from concurrent chemoradiation for new cases and systemic chemotherapy in recurrent/metastatic cases.

Furthermore, adjuvant cetuximab or cisplatin-based-chemoradiation to treat residual disease and regional micrometastasis proved to be an efficient approach [3], despite aggressive primary treatment approaches, locoregional and distant metastatic rates are high with restricted salvage therapies [4], agents that could be used to treat recurrent or metastatic cases include cisplatin, cetuximab, pembrolizumab, nivolumab, taxanes and methotrexate. Several randomised studies comparing progressive disease (PD)-1, and/or PDL-1 inhibitors alone or combined with chemotherapy are ongoing to salvage this group of patients [5, 6].

Reported objective response rates for immune check point inhibitors in recurrent or metastatic SCC fall below 15% with a median overall survival of 6–9 months, however, the addition of chemotherapy could modify immunosuppressive microenvironment of the tumour enhancing the effect of immune check point inhibitors [7, 8]. In two randomised studies, combining cetuximab with systemic chemotherapy in recurrent and metastatic SCC had significantly improved treatment outcomes [9, 10].

Based on EXTREME and TPExtreme studies, cetuximab-based chemotherapy is the treatment of choice for recurrent/metastatic SCC with a median overall survival did not exceed 1 year, despite the great effort to find the optimal chemotherapy combination with cetuximab, still the hope to achieve a long median survival is sorrowful, based on the above dismal results, we aimed from the current study to explore the treatment results of cetuximab in combination with weekly carboplatin and paclitaxel regimen in advanced HNSCC after the failure of radiotherapy and chemotherapy.

## Methods

This study was a non-randomised, single arm, phase 2 efficacy study conducted in two oncology centres in upper Egypt; Clinical Oncology Department of Assiut University and Oncology Department of Assiut Health Insurance, we recruited patients with recurrent inoperable SCC previously treated with surgery ± radiotherapy (RT) or concurrent chemoradiation, participants should be pretreated with platinum whether with RT or in salvage chemotherapy, and should still have adequate auditory and renal functions with adequate BM reserve, also they were at least 18 years of age, they should have a measurable disease per Response Evaluation Criteria in Solid Tumours (RECIST) version 1.1, and received no previous immunotherapy or epidermal growth factor receptor (EGFR) inhibition. PDL-1 testing by immunohistochemistry on biopsy or surgical blocks was a pre-requisite before starting our protocol, performance status (PS) ≤ 3 was allowed, as expected we excluded all cases of advanced nasopharyngeal SCC and metastatic cases. The study was approved by the Ethical Committee of Assiut University (IRB no = 04-2023-300029), informed consent was taken from all participants, and all methodology was carried out in compliance with the Helsinki declaration.

All eligible participants had baseline multislice computed tomography with contrast of head, neck and chest, (and/or magnetic resonance imaging of head and neck with contrast), bone scan, abdominal ultrasound, audiogram evaluation, complete blood picture (CBC) and blood chemistry, moreover, all patients were examined thoroughly to ensure absence of neurological deficits.

Response evaluation was done clinically every week, CBC and blood chemistry were evaluated every other week and imaging was repeated every 9 weeks as long as there was a clinical improvement, and after finishing treatments.

RECIST ver.1.1 was used to determine the response after 9, 18 weeks following the start of protocol on imaging (target lesions were the primary lesion with a length > 1 cm and all LNs with shortest axial diameter ≥ 1.5 cm, other lesions were considered non-target such as LNs < 1.5 cm and thickness < 1 cm, summation of all longest diameters (SLDs) of target lesions were carried out within 4-weeks before the protocol and non-target lesions were evaluated as disappear, stable or progress) by also summating the longest diameters or SLD of target lesion and evaluation of non-target lesions; complete response (CR) defined as complete disappearance of all target and non-target lesions, partial response (PR); defined as ≥30% decrease SLD, no new lesions and no progression of non-target lesions, stable disease (SD); defined as no PR and no PD, PD; ≥20% increase SLD, new lesions or progression of non-target lesions, after imaging, in some cases, narrow band endoscopy with biopsy was done 6–8 weeks after the end of protocol to determine their pathologic response.

Weekly I.V. cetuximab, carboplatin and paclitaxel.

Cetuximab was given at an initial loading dose of 400 mg/m^2^ intravenously in the first week, then weekly 250 mg/m^2^.

Carboplatin dose calculated based on area under the curve = 2.

Paclitaxel dose was 45 mg/m^2^.

Reduction of paclitaxel dose was done in some patients who experienced neuropathy >grade 1. The primary endpoint was overall response rate (ORR) and disease control rate (DCR) defined as CR, PR, and stable responses after 18 weeks of treatment in intention to treat participants, and also, determination of median and 1-year PFS.

Patients achieved adequate downsizing of their tumours, underwent excision of residual lesions ± lymphadenectomy, if possible, also radical dose three-dimensional conformal radiotherapy was given for those who did not receive primary RT.

### Maintenance cetuximab

Patients achieved a response after 18 weeks of treatment (including those who had excision of their residual disease), continued cetuximab alone for 1 year, cetuximab was given every 2 weeks at a dose of 400 mg/m^2^.

Toxicity grades were determined based on common toxicity criteria of adverse events version 4, patients who developed skin rash were treated with doxycycline 100 mg every 12 hours, hydrocortisone cream and antihistaminic.

### Statistics

Data were analysed using IBM-SPSS version 26; descriptive statistics were mean ± SD, median, percentages, numbers and ranges. Inferential statistics in the form of Wilcoxon signed rank test for paired data, likelihood ratio and chi square tests for nominal and ordinal variables, Kruskal–Wallis test for more than two categorical variables and Mann–Whitney *U*-test for two categorical variables were used; Spearman rho correlation was also used for the relation between scale variables.

Kaplan–Meier for graphing survival curves and log rank for comparison of different survival curves between ≥2 categorical variables, all results considered significant at *p*-value ≤ 5%.

Time to tumour progression was defined as the time elapsed from diagnosis to progression on primary treatment.

Progression free survival was defined as the time passed from the start of cetuximab-based treatment to disease progression, death or end of study.

## Results

The current study involved 31 patients with recurrent HNSCC who were recruited over a period of 1 year and then followed up for 2 years, these patients were previously treated with surgery ± adjuvant RT, or concurrent chemoradiotherapy (CCRT) with cisplatin either weekly or on days 1, 22 and 43 of RT, some of the patients (those of oral cavity tumours) had their tumours excised before conformal radiotherapy. Upon failure, they were salvaged with weekly cetuximab, carboplatin and paclitaxel. The median age was 54 years with a male-to-female ratio of 1.4: 1, 21 patients (67.7%) had PS of 0–1, and 45.2% of patients were still smokers (Table 1).

Differentiation of the tumour was documented in 16 patients (G1-2), while 15 patients had grade 3 SCC (48.4%), T3–4 lesions and positive cervical LNs were reported in 67.7% and 64.5% of patients respectively, chemotherapy was given either concurrently with RT, or in neoadjuvant or adjuvant basis in some patients and collectively delivered to 67.7% of patients, the overall positive rate of PDL-1 for 31 patients was 0.52 and distributed as mentioned below (Table 2), furthermore, only 19.4% of patients had surgical excision of their primary tumours, and only 25.8% achieved CR.

**Figure 1.**
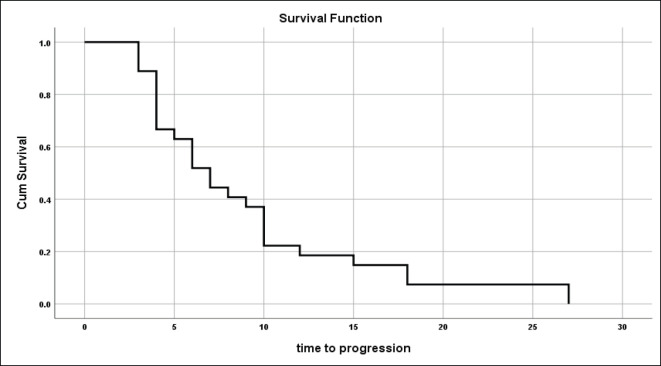
Time to tumour progression for those not progressed on primary treatment.

For those patients with controlled tumours (CR + PR + SD), the median time to disease progression was 7 ± 1.03 months (95% CI = 5–9.02) (Figure 1).

Dynamic changes of tumour sizes in response to 18 weeks of the current treatment protocol.

**Figure 2.**
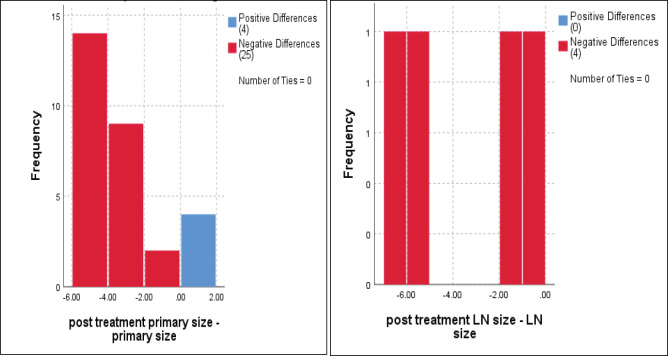
Dynamic reduction of primary tumour size and regional LNs in response to treatments, Wilcoxon signed rank test, *p* < 0.001 and *p* = 0.07, respectively.

The median primary tumour size was 5 cm, and the mean was 6 ± 2 cm (range 3.3–9 cm) which significantly reduced to a median of 1 cm and mean = 2.2 ± 2.8 cm (range 0–10 cm), *p* < 0.001 (Figure 2).

Regarding to regional LNs, only four cases had enlarged cervical LNs at the start of the current protocol, with a median of 3 cm and a mean ± SD = 4.7 ± 2.5 cm, and downsized to 1.5 cm and 1.4 ± 1.1 cm, respectively, *p* = 0.07 (Figure 2).

### Response to the current regimen

Twelve patients (38.7%), 14 (45.16%), 1 (3.2%) and 4 patients (12.9%) achieved CR, PR, SD and disease progression (DP).

**Figure 3.**
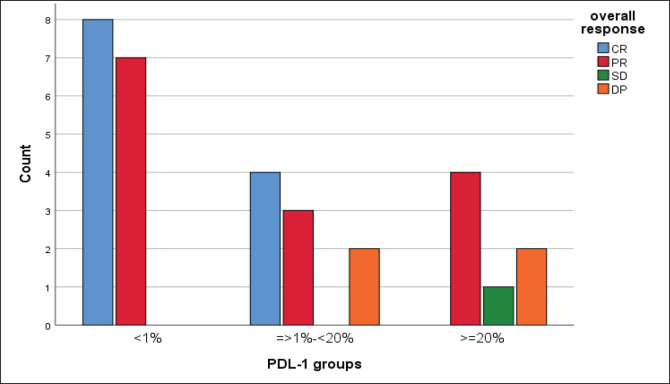
Impact of PDL-1 expression on responses of 31 patients with recurrent HNSCC, likelihood ratio, *p* = 0.02.

**Figure 4.**
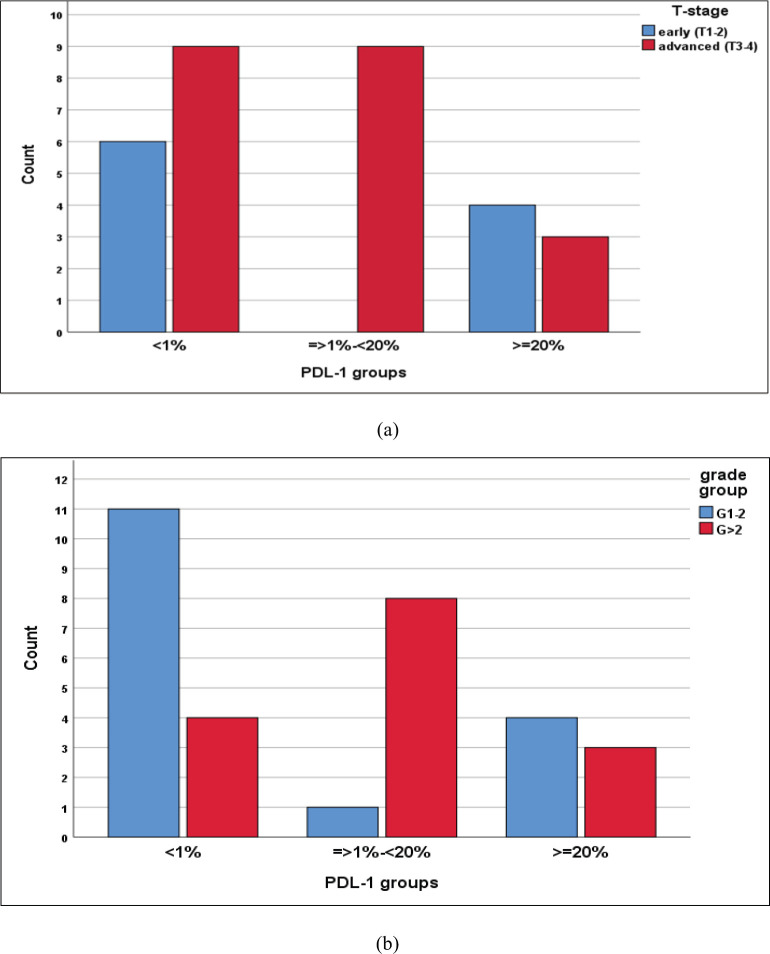
(a): Differences in PDL-1 expression according to T-stage, likelihood ratio, *p* = 0.01. (b): Differences of PDL-1 expression according to tumour grade, likelihood ratio, *p* = 0.008.

We reported a significant impact of PDL-1 expression on response type, where those with CR and PR had lower combined positive score (CPS) score compared with those with SD and DP, *p* = 0.02 (Figure 3), also significant accumulation of PDL-1 expression in advanced T-stage (*p* = 0.01) (Figure 4a), and grade 3 (*p* = 0.008) (Figure 4b). Furthermore, no significant differences in PDL-1 expression according to sex (*p* = 0.6), smoking (*p* = 0.2), tumour site (*p* = 0.3), N-stage (*p* = 0.3) and primary response (*p* = 0.9).

### PFS in recurrent HNSCC

**Figure 5.**
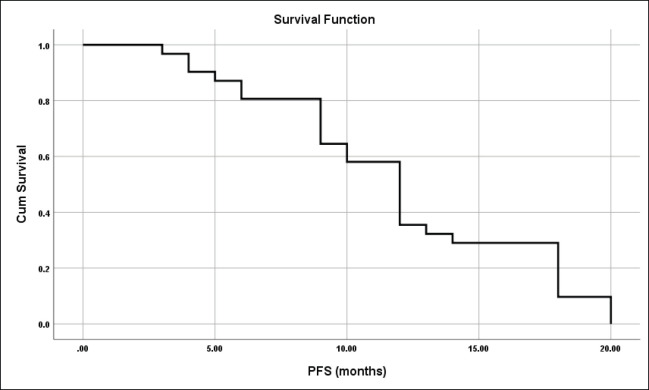
PFS for all studied patients.

The median PFS was 12 months and the mean ± SD was 12 ± 0.92 months (95% CI = 10.2–13.8) (Figure 5).

Seven patients (22.6%) died after a mean duration of 6 ± 2.6 months, 6 patients (19.4%) progressed after a mean duration of 10.2 ± 2.9 months and 18 patients (58.1%) were right censored and still under follow up.

Furthermore, 27 patients completed all 18 weeks of cetuximab-based regimen, while 4 patients progressed after 3–5 months of treatment.

Eighteen patients completed the whole protocol including maintenance therapy, and nine of the remaining patients are currently on the protocol and deaths were reported in progressed patients (seven deaths), final PFS and overall survival (OS) were not reached as patients are still under followup or maintenance.

**Figure 6.**
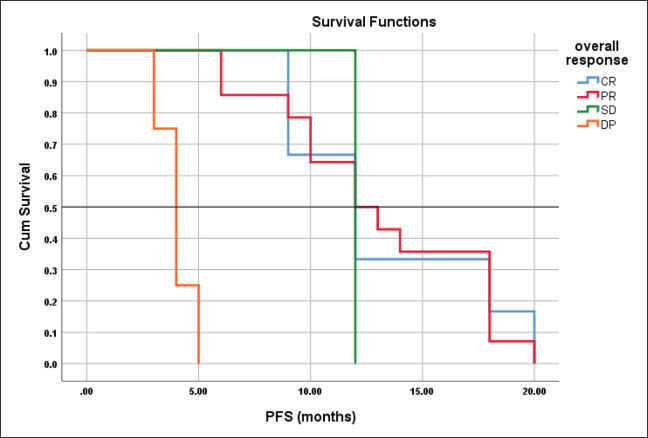
Differences in PFS according to response type.

The mean PFS for those with CR, PR, SD and DP were 13.3 ± 1.3, 13.1 ± 1.2, 12.0, 4.0 ± 0.41 months respectively with the corresponding median PFS were 12-, 12-, 12- and 4-months, log rank = 41.4, *p* < 0.001 (Figure 6).

The ORR (ORR = CR + PR) was 83.9%, DCR (DCR = CR + PR + SD) was 87.1%, ≥1-year PFS was 58.1%, surgical re-excision after cytoreduction was succeeded in ten cases (32.3%) after 18 weeks of treatment, after recovery they were kept under maintenance with cetuximab for an additional year.

**Figure 7.**
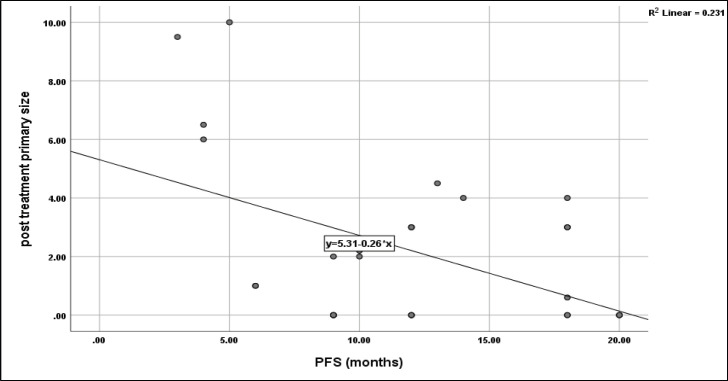
Correlation between post-treatment primary size and PFS, *p* = 0.006.

No significant impact of sex (*p* = 0.1), smoking (*p* = 0.6), grade (*p* = 0.4), primary T (*p* = 0.6), primary N (0.06) and primary response to CCRT (*p* = 0.6) on PFS of the current protocol, however, the primary tumour site had a significant role on PFS (*p* = 0.012) with the highest median PFS was 20 months for oropharyngeal and PNS sites, followed by 12 months for oral cavity and laryngeal sites, and the least was 6 months for hypopharynx. However, PFS was negatively correlated with post-treatment primary tumour size (*r* = −0.48, *p* = 0.006) (Figure 7), while no significant correlation between age and time to progression with PFS (*r* = +0.2, *p* = 0.2 and *r* = −0.14, *p* = 0.5, respectively).

**Figure 8.**
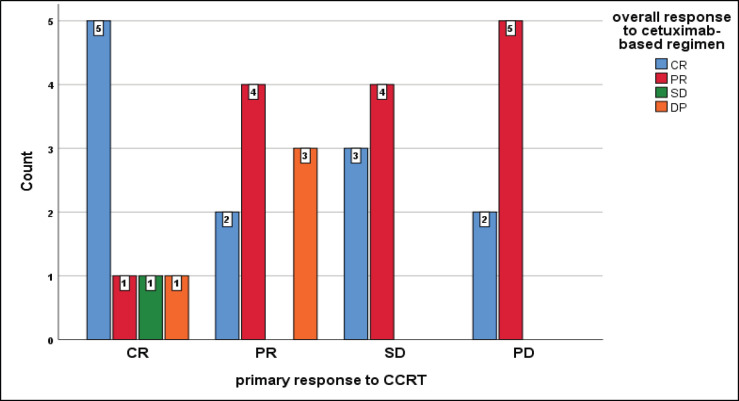
Relation between primary response to CCRT and overall response to cetuximab-based regimen, chi-square test, *p* = 0.2.

Unexpectedly, those patients who achieved primarily DP to CCRT developed CR and PR on cetuximab-based regimen, while all patients who achieved DP on our protocol primarily had CR and PR, furthermore all patients who had a primary SD, developed CR and PR on the current protocol but insignificant (*p* = 0.2) (Figure 8).

**Figure 9.**
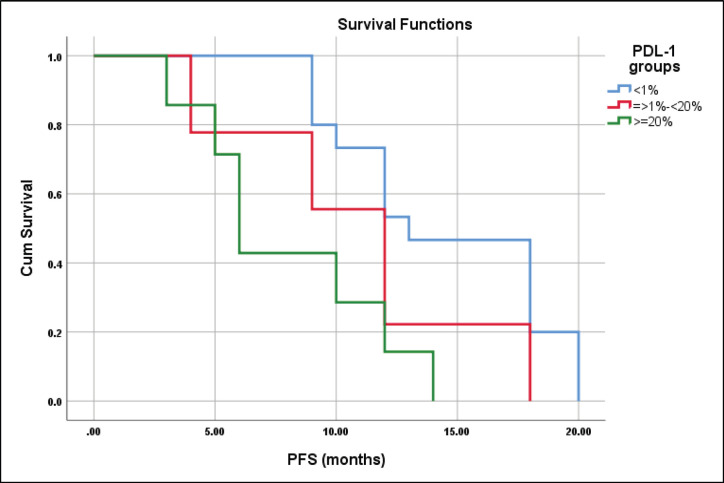
Differences in PFS according to CPS score.

The median PFS for patients with CPS scores <1%, ≥1%–<20%, and ≥20% were 13 ± 1.9, 12 ± 1.2 and 6 ± 0.7 months, respectively, log-rank = 9.3, *p* = 0.01 (Figure 9).

### Toxicity to cetuximab-based regimen

**Figure 10.**
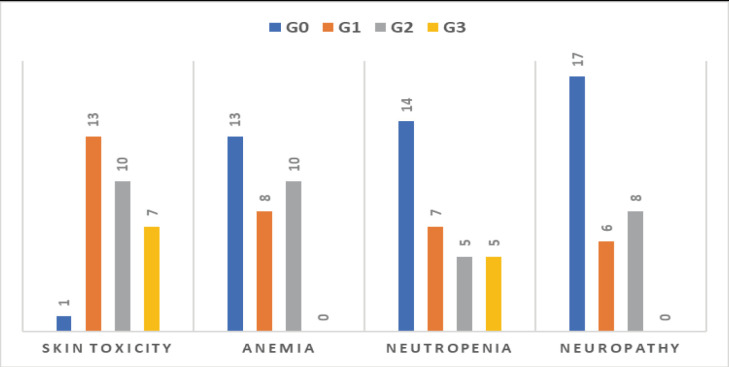
Toxicity among 31 patients treated with cetuximab-based regimen.

The regimen was well tolerated, and all patients were compliant with treatment protocol without compromising grade 3–4 toxicities especially regarding neutropenia, anaemia and neuropathy, but grade 3 skin rash and erythema were developed in 22.6% of patients (7/31) and treated adequately with hydrocortisone cream, antiallergics and doxycycline 100 mg tablet/12 hours in some cases (Figure 10), we did not stop treatment because of intolerance or toxicity.

## Discussion

The incidence of HNSCC is still rising and is anticipated to increase by 30% by 2030 according to GLOBOCAN, which was due to the prevalence of different carcinogens in spite of improvement of 5-year overall survival in the last three decades for all age groups except elderly >75 years, and for all anatomical sites except larynx [11, 12]. The relapse rate is high with multi-modality treatment being the standard of care in many sites, however, patients with recurrent or metastatic disease have a dismal prognosis with a median survival of less than 12 months [13].

Numerous studies have demonstrated aberrant overexpression of EGFR in 80%–90% of HNSCC and associated with resistance to treatment and poor survival [14], so Food and Drug Administration approved molecular targeting of EGFR by monoclonal antibody cetuximab and defined its resistance by overexpression of other tyrosine kinases like HER and MET [15, 16], currently, advanced and recurrent HNSCC are treated with cetuximab along with cisplatin or carboplatin and 5-fluorouracil as a first-line treatment according to EXTREME study that has shown significant improvement of OS compared with chemotherapy alone without cetuximab [17, 18].

Despite the high efficacy of EXTREME protocol in recurrent cases, it was associated with life-threatening adverse effects and poor quality of life, which necessitated the combination of cetuximab with other less toxic chemotherapeutic agents with better safety profile and maintained efficacy [18].

The current study revealed a significant reduction of the size of recurrent primary lesion after 18 weeks of weekly cetuximab, carboplatin and paclitaxel (*p* < 0.001), without a comparable significant reduction of regional LNs (*p* = 0.06), the current ORR was 83.9%, ≥1-year PFS was 58.1%, also surgical intervention was succeeded to salvage 32.3% who did not achieve CR to the current protocol, the median PFS was 12 months which significantly affected by tumour site (*p* = 0.012), PDL-1 expression (*p* = 0.01), and ORR (*p* < 0.001), PFS was compromised by the date of end of study because 58.1% of patients are still alive and either continuing their protocol or finishing it and under follow up, even those patients who died, five of them died due to causes not related to DP or toxicity, while only two died of DP in spite, they died after a duration of 6 ± 2.6 months.

Posch *et al* [19] and Guigay *et al* [20] proved that replacing 5-fluorouracil with taxanes could minimise the toxicity profile and improve the quality of life, cetuximab-containing protocols (63.6% of patients received EXTREME protocol) were retrospectively evaluated in locally recurrent (*N* = 66) and distant metastatic (*N* = 41) HNSCC, PFS for locally recurrent cases was 5.8 months, disease control rate was 62.5% and salvage surgery was associated with longer PFS in recurrent cases [21], despite of being incomparable in demographic data to the previously mentioned study, but comparable in primary tumour site and differentiation status, time to tumour recurrence following RT (46.7% recurred within 6 months compared to 58.1% in our study), however, our protocol achieved a better PFS (12 months compared to 5.8 months).

Furthermore, in Chen *et al* [21], the best ORR was 35.9% in locoregional recurrent cases while for the whole population was 33.1% with only 1 patient achieving CR and 34 patients achieving PR, the current results deviated completely from this alignment, where ORR and DCR were 83.9% and 87.1%, respectively, implying that weekly approach could be a favourable treatment than EXTREME protocol or 3-weekly regimen.

The median PFS outcomes for cetuximab-based treatments in EXTREME, KEYNOTE-048, ENCORE and CHANGE-2 studies [7, 9, 22, 23] were comparable to each other (5.1–6.5 months), which were translated into improvement of OS for locoregional recurrent cases but our results were incomparable to the previous studies and could be attributed to better tolerability of weekly regimen with preferable safety profile allowing us to put these patients on maintenance therapy, a small table for comparison with other studies evaluating immunotherapy-based and cetuximab-based treatments was incorporated to highlight the efficacy of our protocol and safety profile (Table 3). However, a small sample size of the current study interfered with the final conclusion that a weekly regimen could be more efficacious.

Retrospective studies had disclosed favourable outcomes for salvage surgery compared with re-irradiation, and chemotherapy alone [24–27], however, those patients who were salvaged with surgery, had low volume locoregional recurrence and good performance status compared with those who received re-irradiation or chemotherapy, so in the current study, weekly cetuximab, carboplatin and paclitaxel could achieve CR rate of 38.7%, while cytoreduction was achieved in 45.16% of patients sufficiently to be resected in 32.3% of patients.

A meta-analysis of 23 studies including 3,105 patents was analysed and revealed that the overall positive rate of CPS was 0.42 with improved PFS for those with positive PDL-1 expression (*p* = 0.01), contrary to this meta-analysis, the current study had a CPS positive rate of 0.52, and those with CPS <1% had a significantly better PFS (*p* = 0.01) [28]. In addition, high PDL-1 expression was significantly related to clinicopathologic features such as high T stage, and grade 3 which were comparable to Wusiman *et al* [29].

## Conclusion

Based on favourable treatment outcomes, including high ORR and DCR, improved median PFS and tolerable toxicity profile, the current weekly cetuximab, carboplatin and paclitaxel with 1-year maintenance cetuximab in responding patients is considered a feasible and effective regimen, and needs to be evaluated in a multicentric study.

## Conflicts of interest

The authors declared that there were no financial or non-financial competing interests with this work.

## Funding

The authors did not receive any financial support.

## Data availability statement

All data analysed was included within the manuscript.

## Figures and Tables

**Table 1.** Demographic characteristics of study patients.

Demographics	Descriptive
Age (mean ± SD) Median (Min-max)	56 ± 15.8 years 54 years 23–80 years
Sex Male/female	18/13 (1.4: 1)
Performance status PS = 0 PS = 1 PS = 2 PS = 3	8 (25.8%) 13 (41.9%) 7 (22.6%) 3 (9.7%)
Smokers	14 (45.2%)

Data expressed as number, percentages, mean ± SD and median

**Table 2.** Initial clinicopathological characteristics at time of presentation.

Characteristics	Descriptive
Grade of SCC G1 G2 G3	2 (6.5%) 14 (45.2%) 15 (48.4%)
Type of biopsy Endoscopic Punch biopsy TCNB	15 (48.4%) 7 (22.6%) 9 (29%)
Primary site Oral cavity Oropharynx Hypopharynx Larynx Paranasal sinuses (PNS)	13 (41.9%) 2 (6.5%) 9 (29%) 6 (19.4%) 1 (3.2%)
PDL-1 CPS ≤ 1% CPS = ≥1%–<20% CPS ≥ 20%	15 (48.4%) 9 (29%) 7 (22.6%)
Type of previous surgery No surgery Palliative resection Radical resection	25 (80.6%) 3 (9.7%) 3 (9.7%)
Previous chemotherapy	21 (67.7%)
T-stage T2 T3 T4	10 (32.3%) 12 (38.7%) 9 (29%)
N-stage N0 N1 N2 N3	11 (35.5%) 4 (12.9%) 14 (45.2%) 2 (6.5%)
Primary response CR PR SD DP	8 (25.8%) 9 (29%) 7 (22.6%) 7 (22.6%)

Data expressed as number and percentages

Time to tumour progression after primary RT contained treatments

**Table 3.** Comparison between different cetuximab and immunotherapy-based treatments.

Type of treatment	Previous interventions	Outcomes	Adverse events	Compliance
Weekly cetuximab + carboplatin + paclitaxel	CCRT ± surgery	ORR = 83.9% DCR = 87.1% Median PFS = 12 months	Grade 3 skin toxicity = 22.6% (7/31), other adverse events were of grade 1–2	All patients were compliant with treatment protocol. No deaths from adverse events.
Extreme protocol	Recurrent/metastatic cases	ORR = 39% Median PFS = 3.7 months Median OS = 7.2 months	Acute grade 3–4 haematologic and non-haematologic toxicities were 25.2% and 27.2%.	Well tolerated with 29% pain reduction, 13.5% weight gain and 17.2% improved performance.
TPExtreme study compared to EXTREME study	Recurrent/metastatic cases not suitable for curative treatment (271 patients in TPEx versus 270 in EXTREME study)	Median OS was 14.5 months in TPEx versus 13.4 months	Grade ≥3 toxicities were 81% versus 93%, 45% versus 54% developed serious adverse events and 16 versus 21 died of adverse events	Favourable safety profile
Weekly cetuximab, docetaxel and cisplatin	Recurrent/metastatic cases (37 pts)	ORR = 56% mPFS = 4.8 months mOS = 14.7 months	Grades 3 and 4 were 85% and 7%	Tolerable
Keynote 048 Pembrolizumab alone versus pembrolizumab + chemotherapy versus cetuximab + chemotherapy	Recurrent/metastatic cases 301 versus 281 versus 300 patients respectively	Median survival was 11.6 versus 13 versus, 10.7 months	Grade ≥3 was 55% versus 85% versus 83%, deaths due to toxicity were 8% versus 12% versus 10% respectively	Manageable toxicity profile
